# Description of two three-gendered nematode species in the new genus *Auanema* (Rhabditina) that are models for reproductive mode evolution

**DOI:** 10.1038/s41598-017-09871-1

**Published:** 2017-09-11

**Authors:** Natsumi Kanzaki, Karin Kiontke, Ryusei Tanaka, Yuuri Hirooka, Anna Schwarz, Thomas Müller-Reichert, Jyotiska Chaudhuri, Andre Pires-daSilva

**Affiliations:** 1 Forest Pathology Laboratory, Forestry and Forest Products Research Institute, 1 Matsunosato, Tsukuba, Ibaraki 305-8687 Japan; 2 Department of Biology, New York University, 100 Washington Square E., New York, NY 10003 USA; 3 Experimental Center, Medical Faculty Carl Gustav Carus, Technische Universität Dresden, Fiedlerstraße 42, 01307 Dresden, Germany; 4 Buck Institute for Research on Aging, 8001 Redwood Blvd, Novato, CA 94945 USA; 5 School of Life Sciences, University of Warwick, Coventry, UK; 6 Present Address: Division of Parasitology, Faculty of Medicine, University of Miyazaki, Miyazaki, 889-1692 Japan; 7 Present Address: Department of Clinical Plant Science, Faculty of Bioscience and Applied Chemistry, Hosei University, Kajino-cho 3-7-2, Koganei, Tokyo 184-8584 Japan

## Abstract

The co-existence of males, females and hermaphrodites, a rare mating system known as trioecy, has been considered as an evolutionarily transient state. In nematodes, androdioecy (males/hermaphrodites) as found in *Caenorhabditis elegans*, is thought to have evolved from dioecy (males/females) through a trioecious intermediate. Thus, trioecious species are good models to understand the steps and requirements for the evolution of new mating systems. Here we describe two new species of nematodes with trioecy, *Auanema rhodensis* and *A. freiburgensis*. Along with molecular barcodes, we provide a detailed analysis of the morphology of these species, and document it with drawings and light and SEM micrographs. Based on morphological data, these free-living nematodes were assigned to a new genus, *Auanema*, together with three other species described previously. *Auanema* species display convergent evolution in some features with parasitic nematodes with complex life cycles, such as the production of few males after outcrossing and the obligatory development of dauers into self-propagating adults.

## Introduction

Natural selection has favored the evolution of diverse modes of reproduction. Organisms have evolved to reproduce sexually or asexually, self-fertilize or outcross, and exist as either separate sexes or hermaphrodites. One of the biggest challenges in evolutionary biology is to determine how different modes of reproduction evolved^1^. The phylum Nematoda is an excellent group for the comparative study of reproductive modes because it is large and diverse. Although the predominant reproductive mode in nematodes is male/female (dioecy), multiple types of reproduction evolved independently in several clades, including hermaphroditism, parthenogenesis, alternation of hermaphroditism or parthenogenesis with dioecy, and co-existence of males, females and hermaphrodites (trioecy)^2, 3^.

Theoretical models have been developed to clarify the evolutionary steps necessary to change one type of mating system to another. For the evolution of outcrossing to selfing, for instance, a self-fertilization allele should spread rapidly in an outcrossing population because it increases its own transmission^4, 5^. This is counterbalanced by the effect of inbreeding depression, which is the reduced fitness as consequence of accumulation of deleterious recessive alleles. With the incrementally increasing level of inbreeding in a population, selection is predicted to purge deleterious alleles^6, 7^.

Mixed mating strategies, in which organisms reproduce by both self- and cross-fertilization, is a challenging problem for evolutionary biologists^8^. Whether mixed mating systems are evolutionarily stable is still a matter of controversy. According to theoretical models, there are only two stable states in mating system evolution: predominant outcrossing with strong inbreeding depression or predominant selfing with weak inbreeding depression^7, 9^. This assumption led to the suggestion that mixed mating types are transitional and therefore short-lived^7, 10^. However, mixed mating systems seem to persist for long periods of time in some animal groups, even after several speciation events^11^. The type of sex determination system^12^ and the presence of inbreeding depression in a particular species might explain the persistence of a mixed mating type^13^.

A few years ago, two free-living nematode species with a mixed mating strategy were discovered. Both species are trioecious, producing males, females and hermaphrodites. Like the model nematode *Caenorhabditis elegans*, these species have a short life cycle, large number of progeny, and are transparent, which facilitate their use as models for studying the evolution of mating systems. Several of their biological features, such as vulva development^14, 15^, phylogeny^16^, early embryogenesis^17^, ecology^18^, sex determination^19–21^ and mating dynamics^22^ have been studied in the last few years. However, their morphology does not match that of any described rhabditid nematode species, and a formal species description, along with a valid name has not been published. Instead, the species were referred to in publications as isolates SB347 and SB372. We here describe these two new species and present a detailed analysis of their morphology along with molecular barcode sequences. Molecular phylogenetic analyses show that the new species form a separate clade^16^, and they do not fit into any previously defined genus. One clearly related species with three sexes and similar morphology was originally described by Maupas in 1900 as *Rhabditis viguieri* ^23^ and was later placed into the genus *Reiterina* ^24^. However, in molecular phylogenetic analyses, the type species of *Reiterina, R. typica* (Stefánski,1922)^25^, falls into a different clade that is unrelated to the trioecious species^16^. We thus define and describe a new genus, *Auanema*, to accommodate the new species along with *A. viguieri* and two further species with similar phenotypic characteristics.

## Material and Methods

### Nematode material

*Auanema rhodensis* n. sp. (strain SB347) was originally isolated from blood-engorged deer ticks (*Ixodes scapularis*) that were used as bait for nematodes. The ticks were placed in the upper layer of the soil in Kingston (University of Rhode Island), R.I., United States, in September 2001 by E. Zhioua (W. Sudhaus, pers. comm.). Subsequently, a laboratory culture of *A. rhodensis* SB347 was established by W. Sudhaus^14^. *A. freiburgensis* n. sp. (strain SB372) was isolated from a dung pile in Freiburg, Germany, in August 2003 by W. Sudhaus. Both strains have been kept in the laboratory on NGM plates seeded with *Escherichia coli* OP50, as is standard for *C. elegans* ^26^ and preserved cryogenically (e.g. at the NYU Rhabditid Collection).

Both species produce males and females, and hermaphrodites after passage through the dauer stage^19^. The genders were collected separately as follows. In *A. rhodensis*, most female embryos are produced by their mother within the first 15 hours of adulthood^19, 22^. To obtain females, dauer juveniles were placed individually on a small agar plate seeded with OP50 and cultured at 20 °C until adulthood. After these hermaphrodites oviposited 25 eggs or fewer, they were removed. The F1 generation developed into adult females. To obtain hermaphrodites, dauer juveniles were transferred from old cultures onto seeded NGM plates and collected with the Baermann funnel technique after they reached adulthood. Males were hand-picked from 3- to 7-day-old cultures. For *A. freiburgensis*, females were obtained by letting hermaphrodites self-fertilize on individual plates. Most self-progeny under these conditions are either female or male. Hermaphrodites were obtained by isolating dauer juveniles from crowded plates and letting them develop into adults.

### Test crosses

Crosses were performed to test if SB347 and SB372 are biologically distinct species. *A. rhodensis* (strain SB347) and *A. freiburgensis* (strain SB372) females were isolated as described above. For each test cross, one female and one male were placed into a NGM plate seeded with a 3 cm in diameter lawn of *E. coli* O50-1. At least ten crosses between *A. rhodensis* females and *A. freiburgenesis*, and vice-versa, were performed. As controls, we also included intra-strain crosses, which always resulted in cross-progeny.

### Morphological observations

Live material from 3- to 7-day-old cultures was used for morphological observations, photomicrographs and drawings of males and hermaphrodites. The nematodes were mounted on petroleum jelly slides^27^ or on 5% agar pads, and observed, photographed, and drawn with a light microscope (Eclipse 80i, Nikon, Tokyo; or Zeiss Axiophot) equipped with a drawing tube and digital camera system (DXM1200, Nikon Tokyo; or Hamamatsu) and Openlab software (Improvision). Photomicrographs were edited using Adobe Photoshop Elements v.9 (Adobe Systems, San Jose, CA, USA or ImageJ). Type material was prepared according to the methodology previously described^28^. For SEM observation two methods were used. Nematodes from 3- to 7-day-old cultures were fixed overnight in 5% glutaraldehyde in 0.1 M phosphate buffer (pH 7.2), then post-fixed in 0.2 M osmium tetroxide for 90 min, dehydrated in a series of ethanol solutions, and freeze-dried in a desiccation device (JFD-320, JEOL, Tokyo). Dehydrated nematodes were coated with Pt-Pd (80∶20) in an ion-sputter coater for 5 min at the condition of 1.5 KeV and 15 mA. Alternatively, male worms were picked and fixed for at least 48 hrs in 2% paraformaldehyde and 2.5% glutaraldehyde in M9 buffer, dehydrated in a series of ethanol solutions and then critical point dried (CPD 300, Leica, Vienna). Dried worms were coated with platinum using a sputter coater (SCD 050, BAL-TEC, Balzers, Alzenau) with an amperage of 40 mA. In both cases an SEM (S-4800 FE-SEM, Tokyo or JSM 7500 F, JEOL, Garching) operating at 3–5 KV was used to examine the specimens.

### Molecular profiles and phylogeny

Near full-length SSU rDNA and LSU rDNA and part of the largest subunit of RNA polymerase II were amplified from worm lysates of *A. freiburgensis* n. sp. or from reverse transcribed total RNA and sequenced^16^. The resulting sequences were aligned with published sequences of 18 representatives of Rhabditina (see Supplementary Data 1 for Genbank numbers).

The alignment for SSU rDNA was done by hand using secondary structure predictions as reported earlier^16^. LSU rDNA sequences were aligned using ClustalW2 via the EMBL EBI website (http://www.ebi.ac.uk/Tools/msa/clustalw2/). Default parameters were used, but the gap-open-penalty was set to 25 for pairwise and multiple sequence alignment. The resulting alignment was then manually improved. The alignment of RNA Polymerase II sequences was unambiguous.

To test the data for robustness to method of phylogenetic inference, we performed analyses with weighted maximum parsimony (wMP), maximum likelihood (ML) and Bayesian inference (BI). Robustness of the data to character representation was tested using bootstrap and jackknife analyses.

The wMP jackknife analysis was performed with Paup*^29^. A transversion was weighted twice a transition as in previous analyses of rhabditids^16^. The jackknife analysis was run with 1000 replicates and 2 addition sequence replicates in each round.

The ML analysis was run with RAxML ver. 7.2.8 (“BlackBox” version) via the CIPRES Science Gateway on the TeraGrid of NSF^30–34^. A six-parameter substitution model was used with a gamma correction for rate differences across sites (using 25 discrete categories of sites) and a correction for unvarying sites (GTR + G + I). Parameters were estimated from the data. The shape parameter for the gamma distribution of rates was α = 0. 465874. Estimated proportions of nucleotides were: π(A) = 0.262, π(C) = 0.210, π(G) = 0.267, π(T) = 0.261. Estimated rates for the GTR model were: ƒ(AC) = 0.934, ƒ(AG) = 2.431, ƒ(AT) = 1.635, ƒ(CG) = 0.559, ƒ(CT) = 4.591, relative to ƒ(GT) = 1.000.

The BI analysis was run with MrBayes ver. 3.1.2^35, 36^ via the CIPRES portal. A six-parameter substitution model was used with a gamma correction for rate differences across sites and an estimate for the proportion of invariant sites (GTR + G + I). The analysis was run for 2,000,000 generations. Trees and parameters were sampled every 100 generations. Burnin was set to 10% of the samples to calculate the clade credibility values (posterior probabilities) and to estimate the model parameters, which were: π(A) = 0.278, π(C) = 0.217, π(G) = 0.257, π(T) = 0.258. Estimated rates for the GTR model were: ƒ(AC) = 0.737, ƒ(AG) = 1.615, ƒ(AT) = 1.170, ƒ(CG) = 0.622, ƒ(CT) = 3.013, relative to ƒ(GT) = 1.000.

## Results

### Relationships

At least 10 test crosses between one male and one female of each strain (SB372 and SB347) were attempted. Crosses were attempted in both directions (SB372 female and SB347 male, and vice-versa). Although males and females engaged in mating behavior, no embryos were observed in plates. The results show that the strains belong to two different biological species.

**Figure 1:**
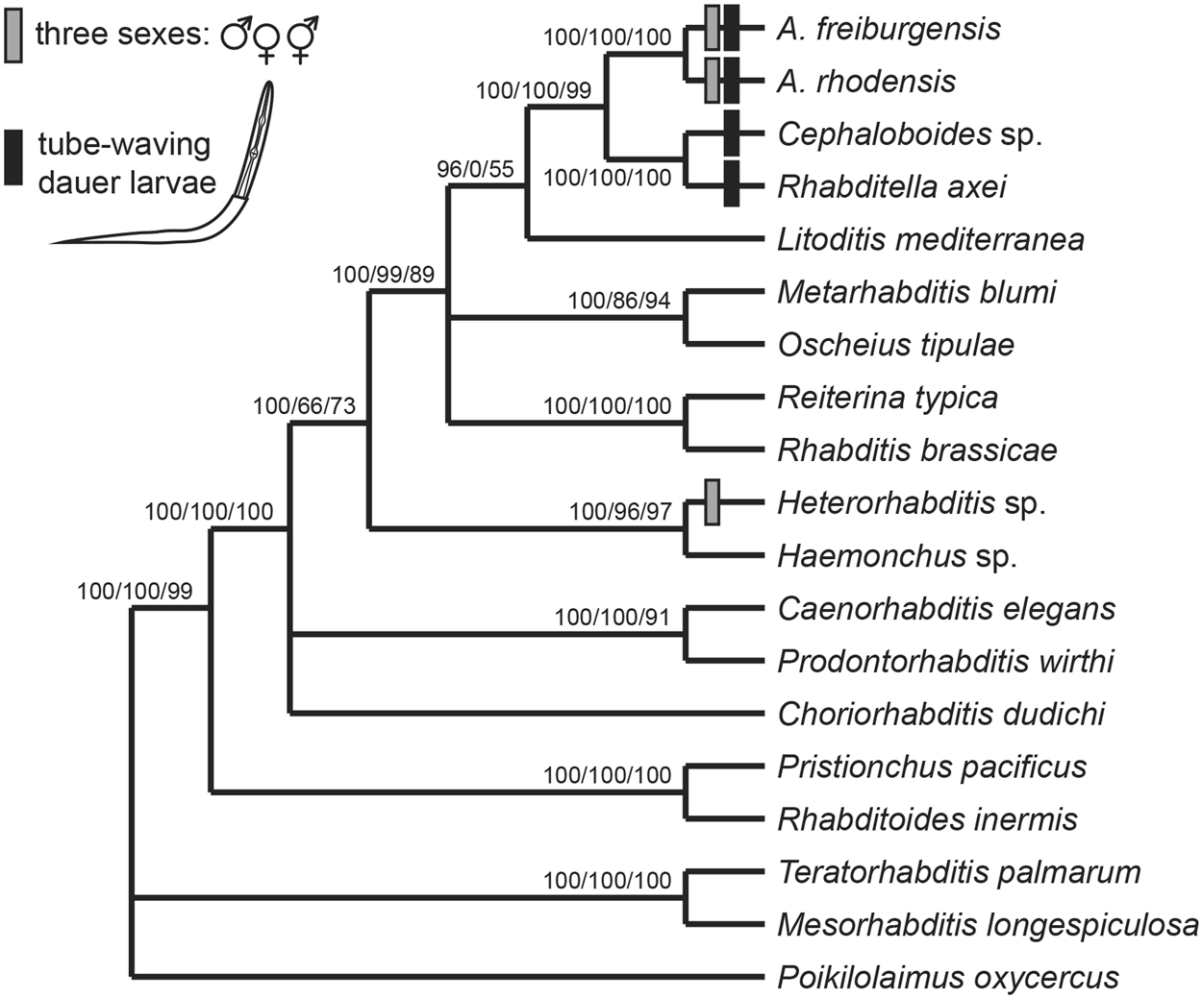
Phylogenetic relationships of the two *Auanema* species and selected Rhabditina based on sequences for near-complete SSU and LSU rDNA and part of the sequence for the largest subunit of RNA polymerase II (*ama-1*). The numbers on the branches show support values in analysis with Bayesian likelihood/maximum likelihood/weighted maximum parsimony (see methods). Species with three sexes or tube-waving dauer juveniles are marked with boxes. The distribution of these characters indicates that a trioecious reproductive system evolved independently in *Auanema* species and *Heterorhabditis*, but tube-waving behaviour evolved only once in the stem species of *Rhabditella, Cephaloboides* and *Auanema*.

The results of our molecular phylogenetic analyses with 19 Rhabditina species (Fig. 1) confirmed the phylogenetic position of SB347 (*A. rhodensis* n. sp.) by Kiontke, *et al*.^16^ and van Megen, *et al*.^37^ (there referred to as *Rhabditis* sp. 4 G) as sister taxon to *Rhabditella* and *Cephaloboides*, and placed SB372 (*A. freiburgensis* n. sp.) as its sister species with 100% statistical support. Phenotypic evidence (see below) suggests that *A. viguieri* is closely related to *A. freiburgensis* n. sp. and *A. rhodensis* n. sp. Sudhaus^24^ had placed *A. viguieri* in the genus *Reiterina*. However, *Reiterina* (represented by *R. typica*) is not related to the two new species in the phylogeny by Kiontke, *et al*.^16^ and in our new analyses with three different algorithms. This was our motivation to establish the new genus *Auanema* and transfer *A. vigueri* as well as two other phenotypically similar species into this genus: *A. reciproca* ^38^ and *A. seurati* ^39^. In the most recent taxonomic treatment of Rhabditina^24^ the former species was retained in the genus *Rhabditis* and the latter was transferred to the genus *Reiterina*. The taxonomic placement of these species, which are known only from the literature, relies on phenotypic similarities. Our arguments for transferring them into *Auanema* are detailed below (Differential Diagnosis section). The sister group relationship of *Auanema* and *Rhabditella* plus *Cephaloboides*, as it emerges from the molecular phylogenies, is supported by an unusual phenotypic character, i.e. the tube-waving behaviour of the dauer juveniles, which was described for *A. reciproca* by Sudhaus (1974), is a common character in *Rhabditella* and was also observed in a *Cephaloboides* species^40^. We hypothesize that this feature evolved in the stem species of *Auanema*, *Cephaloboides* and *Rhabditella* and is thus an apomorphic character of a clade uniting these three genera. Furthermore, our phylogenetic analysis confirms that *Auanema* species with three sexes are not closely related to *Heterorhabditis*, another clade with males, females and hermaphrodites, supporting the notion that this reproductive mode evolved twice convergently within Rhabditina.

### Definition of the genus *Auanema* n. gen

The generic epithet ‘áua’ of this new genus is derived from ‘hair’ in Tupi, an indigenous language from South America.

#### Rhabditidae

Diagnostic characters of the genus that are in this combination not found in species from other genera: Males much smaller than females/hermaphrodites. Stoma long, stegostom comprises about half of stoma (pharyngeal sleeve present), ornaments on metastegostom indistinct; median bulb well developed, round to square. Female tail conical; bursa on male tail open, peloderan or leptoderan with more or less distinctly bilobed posterior margin; 8 genital papillae and papilliform phasmids posterior to GPs. One or two GP precloacal, the 5^th^ and 7^th^ attached to the dorsal surface of the fan. The 6^th^ GP separate, the 7^th^ and 8^th^ close together. Spicules separate and dagger-shaped; gubernaculum half as long as spicules. Dauer juveniles exhibit tube waving behavior. 3 sexes may be present: males, females and hermaphrodites. Males are generally rare.

Type species: *Auanema rhodensis* n. sp.

Valid species in *Auanena* (five):

*A. freiburgensis* n. sp.

*A. reciproca* (Sudhaus, 1974) n. comb.

*A. rhodensis* n. sp.

*A. seurati* (Maupas, 1916) n. comb.

*A. viguieri* (Maupas, 1900) n. comb.

## Species descriptions for ***A. freiburgensis*n. gen. n. sp. and*A. rhodensis*n. gen. n. sp**. 

The two species described here are morphologically similar, especially in female and hermaphrodite morphologies. Therefore, morphology common to both species is described first, followed by species-specific characters and diagnoses for each species.

### Common characters

#### Adults

Body cylindrical. Cuticle with fine annulation. Lateral field inconspicuous, only weakly separated from the other part of body surface by lack of annules, ridges (alae) absent. Lip region not clearly offset, with six equal-sized sectors, two dorsal sectors, right lateral and subventral sectors and left lateral and subventral sectors are close to each other and form a somewhat triangular stomatal opening. Each lip with a setiform labial sensillum. Four setiform cephalic sensilla present. Ampids oval-shaped pores at the level of the posterior end of cheilostom. Stoma cylindrical, separated into three elements, cheilostom, gymnostom and stegostom. Cheilostom and gymnostom simple cylinder, margin of arcade syncytia is faintly visible at the middle of gymnostom. Pro- and meso-stegostom simple cylinder, comprising a little more than half of the stomatal tube (form pharyngeal sleeve); meta-stegostom slightly anisotopic and isomorphic with one small denticle on each sector, slightly more posterior on the dorsal side. Procorpus cylindrical; metacorpus forming a well-developed median bulb, isthmus slender, basal bulb rounded (not polygonal) with weak duplex haustrulum posterior to valves. Procorpus plus metacorpus slightly longer than isthmus plus basal bulb. Cardia (pharynx-intestine junction) conspicuous. Nerve ring surrounding the posterior part of isthmus. Excretory pore conspicuous in ventral and lateral views, variable in position among individuals. Excretory tube extends anteriorly, and then reflexes to continue posterior. An excretory cell is observed a little posterior to the excretory pore opening. Deirid at the same level with excretory pore.

#### Adult male

Tail region slightly ventrally curved when killed by heat. Testis single, on the right of intestine; anterior part ventrally reflexed. Distal part of gonad is usually empty or contains small sperm cells; this part is interpreted as *vas deferens*. Two subventral and one dorsal cloacal (anal) glands visible at the level of anterior end of retracted spicules. Spicules paired, forming a “V” shape in ventral view; smoothly arcuate in lateral view, often protracted in heat-killed specimens. Gubernaculum short, oval in ventral view. Bursa leptoderan in *A. rhodensis* and peloderan in *A. freiburgensis*, anteriorly open. Eight pairs of genital papillae (GP) present. Two pairs of GP located precloacally, one pair adcloacally. Tips of GP5 and GP 7 attached to the dorsal side of bursa, all other GPs are attached ventrally. Papilliform phasmids with ventral openings in a terminal position near the tail tip.

#### Adult female and hermaphrodite

Females and hermaphrodites are morphologically indistinguishable. Body weakly, smoothly and ventrally arcuate when heat-relaxed. Vulva located at mid body, forming horizontal slit 1/3 of vulval body diam. in length; cuticle around the vulva lacks annulations. Gonads didelphic and dorsally reflexed, anterior and posterior gonads on the right and left of intestine respectively; the germ cells arranged in two to three rows in distal half, well-developed oocytes arranged in a single row in the other part; the most developed oocytes clearly darker than the other germ cells. Oviduct connecting ovary (ovotestis) and spermatheca, formed by small rounded cells. Spermatheca and uterus with thick wall. Dorsal wall of the junction of anterior/posterior uterus thickened. Vagina perpendicular to body surface, possessing thick wall, constricted by sphincter muscle at the vagina-uterus junction. Young females/hermaphrodites carry usually none or only one embryo in each uterus, in old individuals, many (more than 10) well-developed embryos are present in an expanded uterus, and the other gonadal structure become vague. Two subventral and one dorsal rectal glands observed surrounding intestine-rectum junction and anterior part of rectum. Rectum as long or slightly longer than anal body diam. Anal opening a horizontal slit; posterior anal lip expands slightly in lateral view. Tail smoothly tapered to finely elongated conical tip but not filiform.

#### Dauer juveniles

Body cylindrical, straight or weakly ventrally arcuate when heat-relaxed. Cuticle thin, smooth, coarsely and shallowly annulated, with two lines of conspicuous alae. Anterior end dome-shaped, continuous with body. Amphids oval-shaped pore, conspicuous, at the level of the posterior end of cheilostom. Labial sensilla observed as refractive dots, inconspicuous. Initially ensheathed in coarsely annulated J2 cuticle; the border between J2 cuticle and dauer body seems transparent. J2 cuticle is attached to substrate when dauer juvenile begins to wave; then, J2 cuticle serves as a protective tube. Stoma narrow, cylindrical, weakly sclerotized, anterior end closed; separation among cheilostom, gymnostom and stegostom not clear, but pharyngeal sleeve which indicates the stegostom is observed. The posterior end of stoma (meta- and telostegostom) more sclerotized compared with the other stomatal elements. Procorpus narrow, cylindrical, not well-developed. Metacorpus forming weak median bulb. Isthmus, slender. Basal bulb with duplex haustrulum posterior to valves, smoothly connected to cardia. Procorpus plus metacorpus slightly longer than isthmus plus basal bulb. Cardia (pharynx-intestine junction) conspicuous, funnel-shaped. Nerve ring surrounding the middle or posterior part of isthmus. Excretory pore opening ventral at the level of basal bulb, conspicuous in ventral and lateral views. Excretory tube extends anteriorly, and then reflexes posteriorly. Excretory cell observed a little posterior to excretory pore opening. Genital anlagen visible ventrally at mid-body; cells are linearly arranged, but the number of cells was not clearly observed. Two subventral and one dorsal glands observed at intestine-rectum junction and anterior part of rectum. Tail conical with bluntly pointed tip.

### *Auanema rhodensis* n. sp

**Figure 2:**
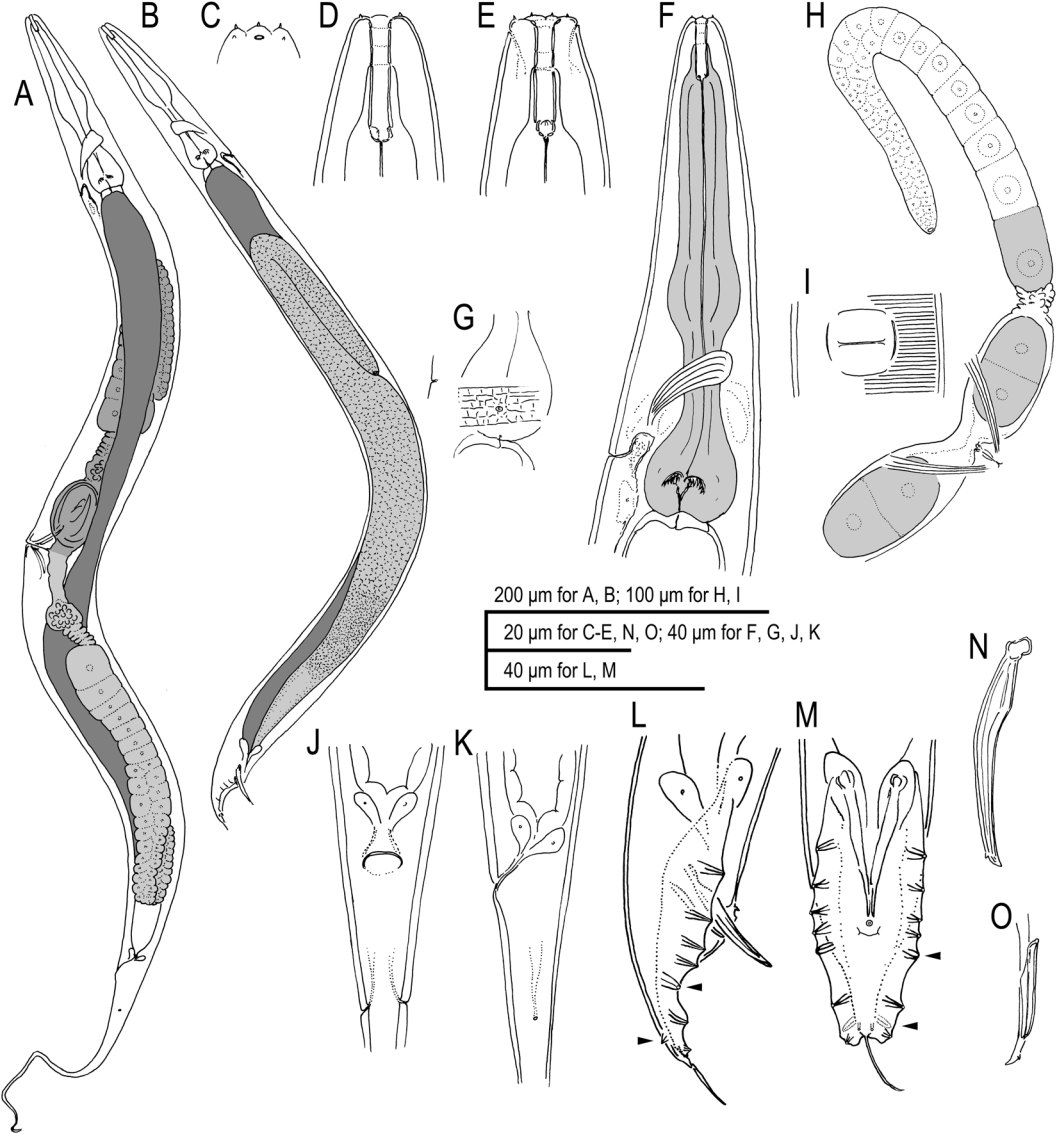
Adult male and hermaphrodite of *A. rhodensis* n. gen., n. sp. (**A**) Hermaphrodite; (**B**) Male; (**C**) Surface of lip region; (**D**) Stomatal region in left lateral view; (**E**) Stomatal region in ventral view; (**F**) Anterior part of hermaphrodite in left lateral view; (**G**) Surface structure with deirid showing relative position to basal bulb and excretory pore; (**H**) Anterior gonad and vulval region of mature hermaphrodite; (**I**) Ventral view of vulval opening in mature hermaphrodite; (**J**) Anal region of hermaphrodite in ventral view; (**K**) Anal region of hermaphrodite in left lateral view; (**L**) Male tail in right lateral view (dorso-laterally directed papillae are indicated by arrowheads); (**M**) Male tail in ventral view (dorso-laterally directed papillae are indicated by arrowheads); (**N**) Spicule in right lateral view; (**O**) Gubernaculum and spicule tip in left lateral view.

**Figure 3:**
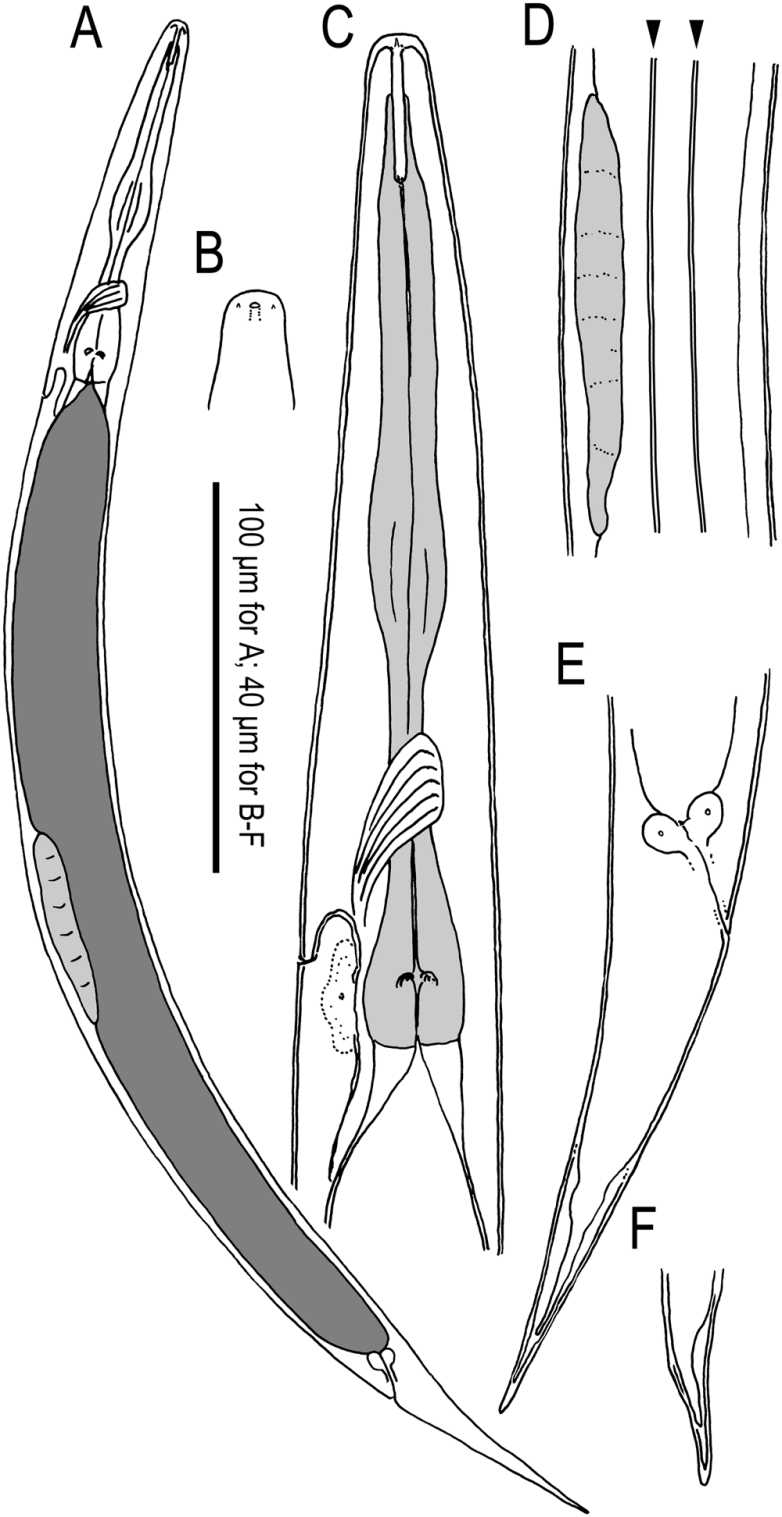
Dauer juvenile of *A. rhodensis* n. gen., n. sp. (**A**) Whole body in left lateral view; (**B**) Surface of lip region; (**C**) Anterior part in left lateral view; (**D**) Genital anlage and lateral field with alae on body surface (indicated by arrowheads): (**E**) Tail region in right lateral view; (**F**) Variation in tail tip in right lateral view.

Figures 2–3, Supplementary Figs S1–S4, Supplementary Data 2 (videos showing z-stacks of photomicrographs for selected morphological features of males, females and hermaphrodites).  = *Rhabditis* sp. SB347 in Denver, *et al*.^3^, Félix^14^, Kolotuev and Podbilewicz^15^, Kiontke, *et al*.^16^, Brauchle, *et al*.^17^, Félix and Duveau^18^, Chaudhuri, *et al*.^19^, Tandonnet and Pires-daSilva^20^, Shakes, *et al*.^21^, Chaudhuri, *et al*.^22^, Pires-daSilva and Parihar^41^, Fabig, *et al*.^42^, Barrière and Félix^43^, Félix and Barkoulas^44^, Félix^45^, Streit^46^, Kulkarni, *et al*.^47^. = SB347 in Kiontke and Fitch^48^.

### Measurements

See Table 1 and Supplementary Table S1.

**Table 1:** Comparison of selected features of five *Auanema* n. gen. species F = female, H = hermaphrodite, M = male; GP = genital papilla; v = papillae attached ventrally on bursal velum, d papillae attached dorsally.

Character	*rhodensis* n. sp.	*reciproca*	*seurati*	*freiburgensis* n. sp.	*viguieri*
body length male (µm)	595–737	521–735	455–755	625–705	715
body length female morph (µm)	980–1214 (F) 767–1401 (H)	768–1098	1155–1500	969–1110 (F) 977–1135 (H)	1330
male much smaller than female	yes (60%)	yes (~66%)	yes (<50%)	yes (~65%)	yes (53%)
cuticle	very thick	very thick	thick	thin	very thin
cuticle structure	vertical lines between annules	?	?	No vertical lines between annules	?
lips in three pairs of two	yes	?	?	yes	yes
stoma length female morph (µm)	12–16	16–19	?	15–18	20
Stoma ratio l/w	9.3 (M), 6.5 (F), 6.6 (H)	~11 (M), 8–9 (F)	?	8.5 (M), 6.4 (F), 7.4 (H)	~6
cheilost./gymnost./stegost. ratio (F)	1:3:5	1:2:4 (figure)	?	1:2:4	~1:2:4 (figure)
stoma slightly anisotopic	yes	yes	?	yes	?
stegostom (sleeve) in % stoma length	average 56 (M), 57 (F), 59 (H)	52–66	?	average 59 (M), 57 (F), 58 (H)	~60
structure on metastegostom	inconspicuous	absent or inconspicuous	?	inconspicuous	?
spicule head	square	square	?	round	round
spicule tip with dorsal thorn	yes	?	?	yes	?
spicule length (µm)	29–32	27–31	?	21–23	23
gubernaculum/spiculum	45%	38–46%	?	49%	50%
bursa distal flaps	round	round	?	pointy or triangular	round, shallow
Male tail	leptoderan	leptoderan	peloderan, sometimes leptoderan	peloderan	peloderan
ray arrangements: distances between rays in comparison	v1-v2 = v2-v3 ad-v5 > v2-v3	v1-v2 = v2-v3 ad-v5 > v2-v3	?	v1-v2 > v2-v3 ad-v5 = v2-v3	v1-v2 ≫ v3-v3 ad-v5 > v2-v3
number of bursal papillae	8GP + ph	8GP + small ph	8	8GP + ph	9
GPs precloacal	2	2	2	2	1
ad, pd GP in position	5, 7	5, 7	?	5, 7	?
phasmids papilliform	yes	yes	?	yes, short	probably
Vulva position relative to body length (V)	48%	46–49%	anterior of middle	48%	~50%
#eggs in uteri (young animals)	1–2	6–13	< 6	1–2	≤ 4
egg size (µm)	39–48 × 24–32 (F) 43–54 × 22–37 (H)	48–56 × 30–34	50 × 27	42–51 × 14–21 (F) 58–61 × 29–38 (H)	45–55 × 28 × 32
a (L/maxW) female morph	13.7–22.6 (F) 13.8–21.5 (H)	18.8–21.4 (F) 14.4–18.5 (H)	17–24	16.2–18.5	28.9
b (length/pharynx) female morph	6.9–8.7(F), 5.8–7.8 (H)	5.2–7.5	7–8	7.8–9.1(F), 7.4–8.5 (H)	7.7
c (length/tail) female morph	5.1–6.3(F) 4.3–12.2 (H)	5.2–6.9	6–7.5	5.4–5.9(F) 5.1–6.0 (H)	4.8
waving of dauer juveniles	tube wavers	tube wavers	?	tube wavers	tube wavers
reproductive mode	H, M, F	described as parthenogenic no sperm was seen in females	described as hermaphroditic Males rare	H, M, F	H, M, F
locality	Kingston (RI), also France	Germany	Algeria	Freiburg (Germany)	Algeria
habitat	ticks used as bait; often in rotting organic material	unknown	diseased flower bulb	dung, mostly horse	rich soil

### Description

#### Adults

Common characters as described above. Cuticle very thick with fine annulation and vertical lines between annules that are observed only by LM, not by SEM; annuli about 1.5–2.0 μm wide at mid-body. Stoma cylindrical, separated into cheilostom, gymnostom and stegostom with roughly 1/9, 1/3 and 5/9 of total stomatal length, respectively; thus, pharyngeal sleeve comprises between 52 and 60% of stoma length. Position of excretory pore variable at a level between the posterior part of basal bulb and a little posterior to cardia.

#### Adult male

Common characters as described above. Stoma on average 9 times as long as wide. *Vas deferens* (see above) comprises distal 1/7 of gonad. Spicule with square manubrium; blade widest at the 1/3 total spicule length then smoothly tapered to bluntly pointed tip; a small but pronounced dorsal spike-like projection present a little proximal of spicule tip. Gubernaculum, narrow in lateral view, a little less than 1/2 of spicule in length; tape-like extensions at of both sides cover the dorsal side of spicule blade. Bursa leptoderan, somewhat polygonal, with smooth edges; distal end of bursa deeply notched, forming a rounded flap on each sides of a short and slender tail spike. The eight pairs of genital papillae (bursal rays) arranged as < GP1, GP2, (GP3, CO), GP4, GP5d, GP6, (GP7d, GP8) phasmid > , where, the distances between GP1-GP2, GP2-GP3 and GP6-GP7 are similar to each other, the distance of GP5d- GP6 clearly larger and that of GP3-GP4 and GP4-GP5d shorter; GP7d and GP8 are close to each other.

#### Adult female and hermaphrodite

Common characters are as described above. Stoma 6–7 times as long as wide. Rectum slightly shorter than anal body diam. A pair of phasmids located laterally at 1.2–1.6 anal body diam. posterior to anus.

#### Dauer juveniles

Common characters are as described above. Rectum as long as anal body diam.

### Type host and locality

The species was originally isolated from ticks (*Ixodes scapularis*) placed on soil as baits for nematodes in Kingston, R.I., United States in September, 2001 by Elyes Zhioua, and was established as laboratory culture by Walter Sudhaus.

### Type material

Holotype male and paratype males were collected from a 6-day-old culture of the type strain SB347 on a NGM agar medium seeded with *E. coli* OP50. The paratype females were collected from the first 25 offspring of hermaphrodites as described in Material and Methods. The paratype hermaphrodites were collected from a 3-day-old culture on a NGM agar medium seeded with *E. coli* OP50.

The holotype male and four paratype males, five paratype females and five paratype hermaphrodites are deposited in the USDA Nematode collection, Beltsville, MA, USA; five paratype males, five paratype females and five paratype hermaphrodites are deposited in the Forest Pathology Laboratory Collection of FFPRI, Tsukuba, Japan.

### *Auanema freiburgensis* n. sp

**Figure 4:**
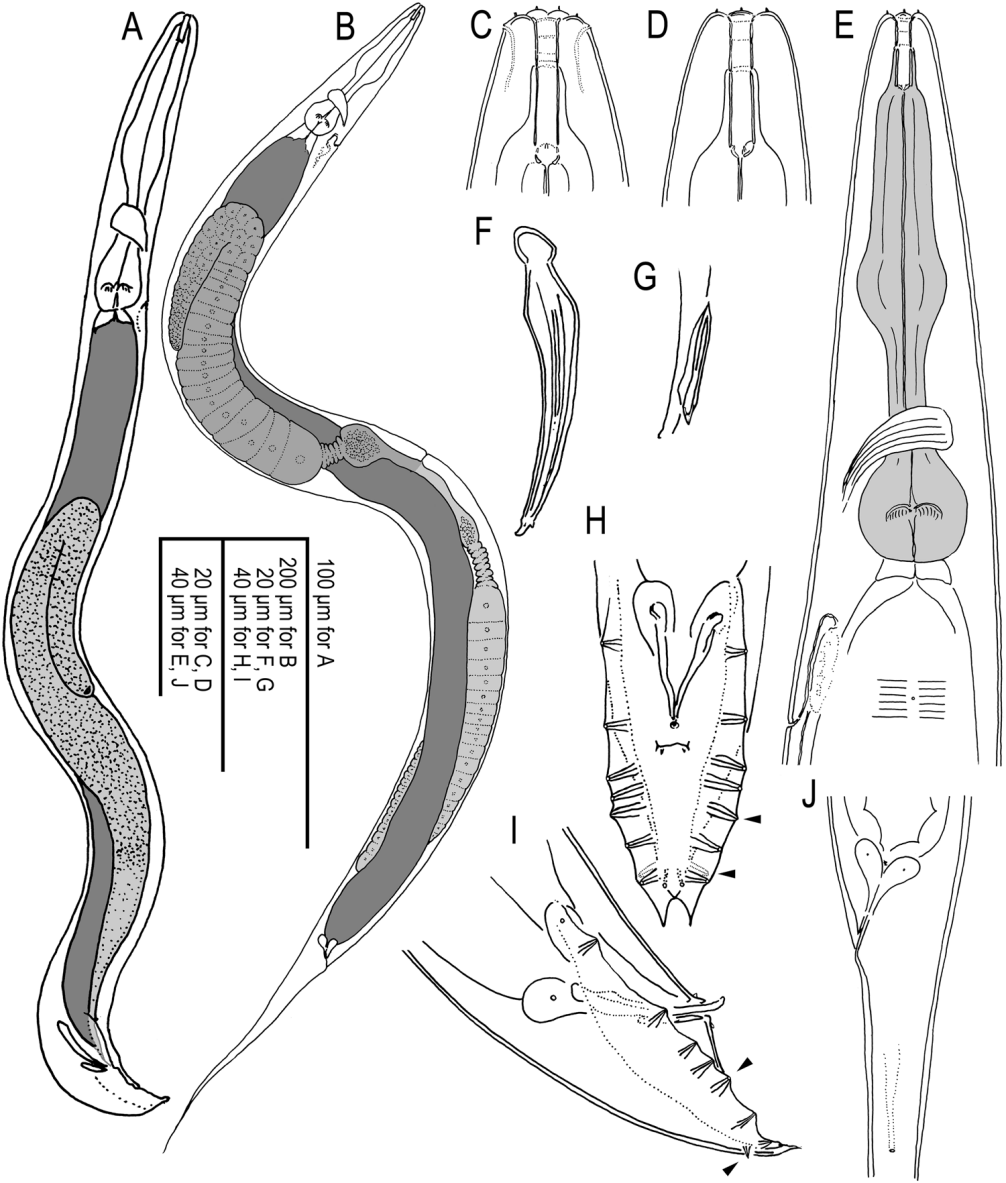
Adult male and hermaphrodite of *A. freiburgensis* n. gen., n. sp. (**A**) Male; (**B**) Hermaphrodite; (**C**) Stomatal region in ventral view; (**D**) Stomatal region in left lateral view; (**E**) Anterior part of hermaphrodite in left lateral view with surface structure and deirid; (**F**) Spicule in left lateral view; (**G**) Gubernaculum in left lateral view; (**H**) Male tail in right lateral view (dorso-laterally directed papillae are indicated by arrowheads); (**I**) Male tail in ventral view (dorso-laterally directed papillae are indicated by arrowheads); (**J**) Anal region of hermaphrodite in left lateral view.

**Figure 5:**
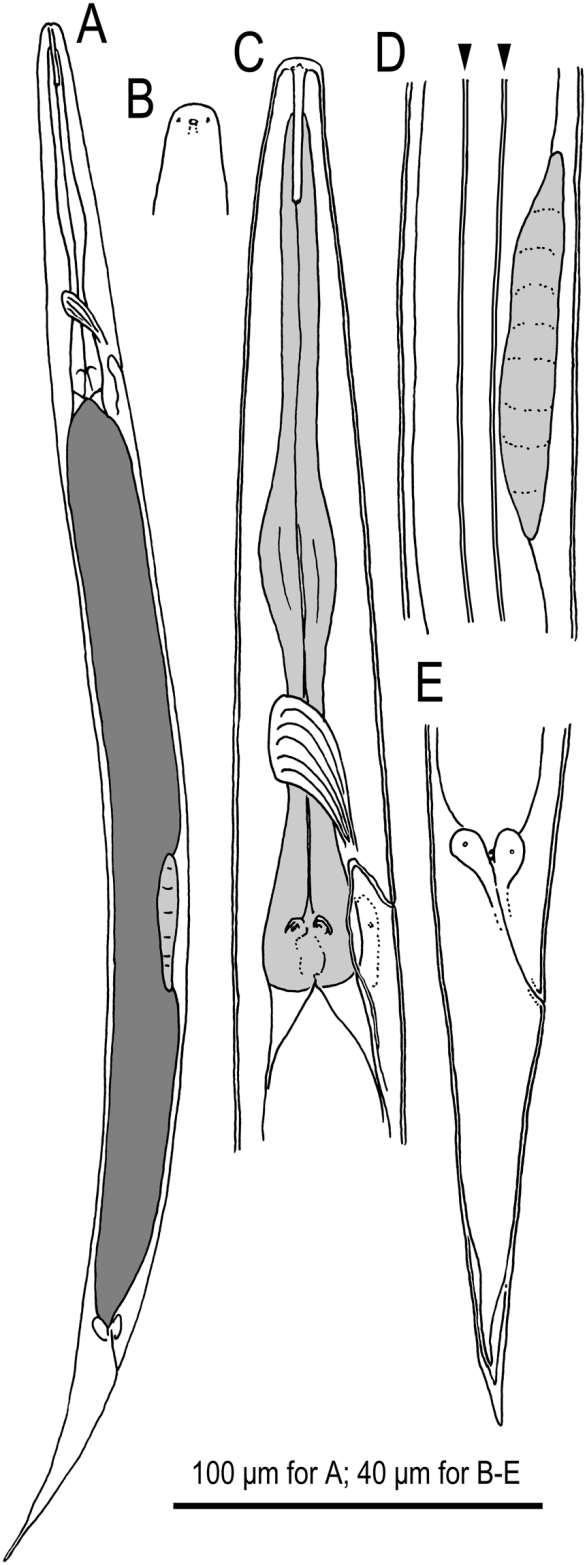
Dauer juvenile of *A. freiburgensis* n. gen., n. sp. (**A**) Whole body in right lateral view; (**B**) Surface of lip region; (**C**) Anterior part in left lateral view; (**D**) Genital anlage and lateral field with alae on body surface (indicated by arrowheads): (**E**) Tail region in right lateral view.

**Figure 6:**
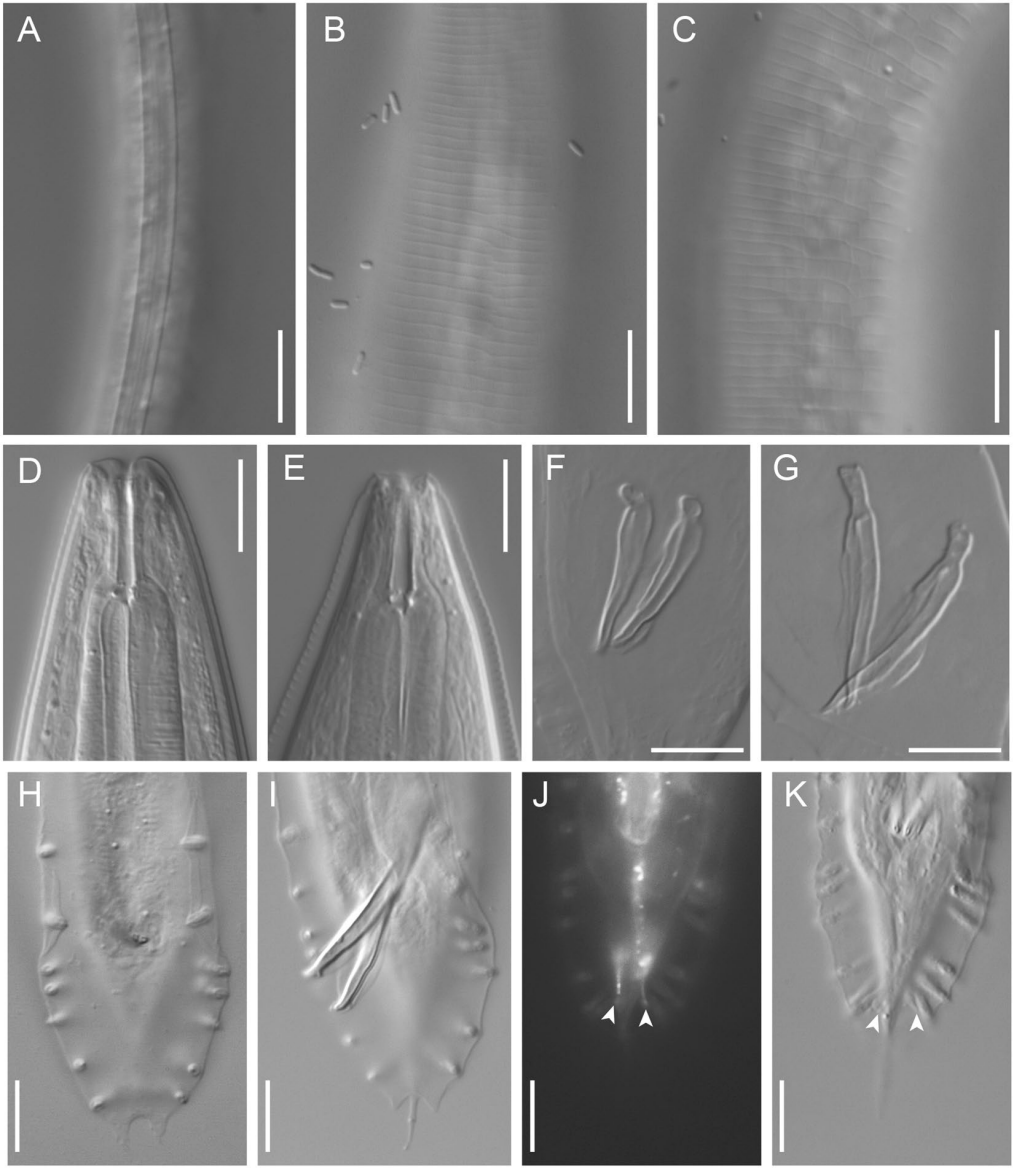
Selected characters of *A. freiburgensis* n. gen., n. sp. and *A. rhodensis* n. gen., n. sp. in comparison: Lateral field of a *A. freiburgensis* dauer juvenile (**A**) and hermaphrodite (**B**) and a *A. rhodensis* hermaphrodite (**C**); note the absence of alae in adults of both species. Stoma of a *A. freiburgensis* hermaphrodite in right lateral view (**D**) and a *A. rhodensis* hermaphrodite in left lateral view (**E**). Spicules of *A. freiburgensis* (**F**) and *A. rhodensis* (**G**). Male tail in ventral view in *A. freiburgensis* (**H**) and *A. rhodensis* (**I**–**K**). J and K show a male in which the phasmids (arrowheads) were stained with FITC and observed under fluorescent light (J) and DIC (K). All scale bars 10 µm.

Figures 4–6, Supplementary Figs S4–S5, Supplementary Data 2 (videos showing z-stacks of photomicrographs for selected morphological features of males, females and hermaphrodites).

### Measurements

See Table 1 and Supplementary Table 1.

### Description

#### Adults

Common characters as described above. Cuticle thin with fine annulation, annuli about 1.5–2.0 μm wide at mid-body. Stoma cylindrical, *ca* 7 times longer than wide, separated into three elements, cheilostom, gymnostom and stegostom with roughly 1/7, 2/7 and 4/7 of total stomatal length, respectively. Position of excretory pore variable at the level from the posterior part of basal bulb to one maximum body diameter posterior to cardia.

#### Male

Common characters are as described above. Stoma on average 8 times as long as wide. *Vas deferens* (see above) comprises distal 1/5 of gonad. Spicule with rounded manubrium; blade widest at the 1/5 total spicule length; then smoothly tapered to blunt tip with small dorsal round projection. Gubernaculum, narrow in lateral view, 49% of spicule length; tape-like extensions on both sides cover the dorsal side of spicules. Bursa open peloderan, with smooth edge, covering whole tail region from the level of the anterior end of retracted spicule; bursa deeply notched distally, forming two triangular flaps. Eight pairs of genital papillae arranged as < GP1, GP2, (GP3, CO), GP4, GP5d, GP6, (GP7d, GP8) > , where the distance between GP1-GP2 is large. A pair of papilliform phasmids opens ventrally near the tail tip, but are sometimes difficult to observe.

#### Female and hermaphrodite

Common characters are as described above. Stoma 6–8 times as long as wide. Rectum almost as long as anal body diam. A pair of phasmids located laterally at 1.4–2.0 anal body diameter posterior to anus.

#### Dauer juveniles

Common characters are as described above. Rectum *ca*. 1.5 anal body diameter in length.

### Type host and locality

The species was isolated from a dung pile (different dung, mostly from horses) in Freiburg, Germany in August 2003 by Dr. Walter Sudhaus.

### Type material

Holotype male and paratype males, females and hermaphrodites were isolated as described in materials and methods. The holotype male and four paratype males, five paratype females and five paratype hermaphrodites are deposited in the USDA Nematode collection, Beltsville, MA, USA; five paratype males, five paratype females and five paratype hermaphrodites are deposited in the Forest Pathology Laboratory Collection of FFPRI, Tsukuba, Japan.

### Differential diagnosis

**Figure 7:**
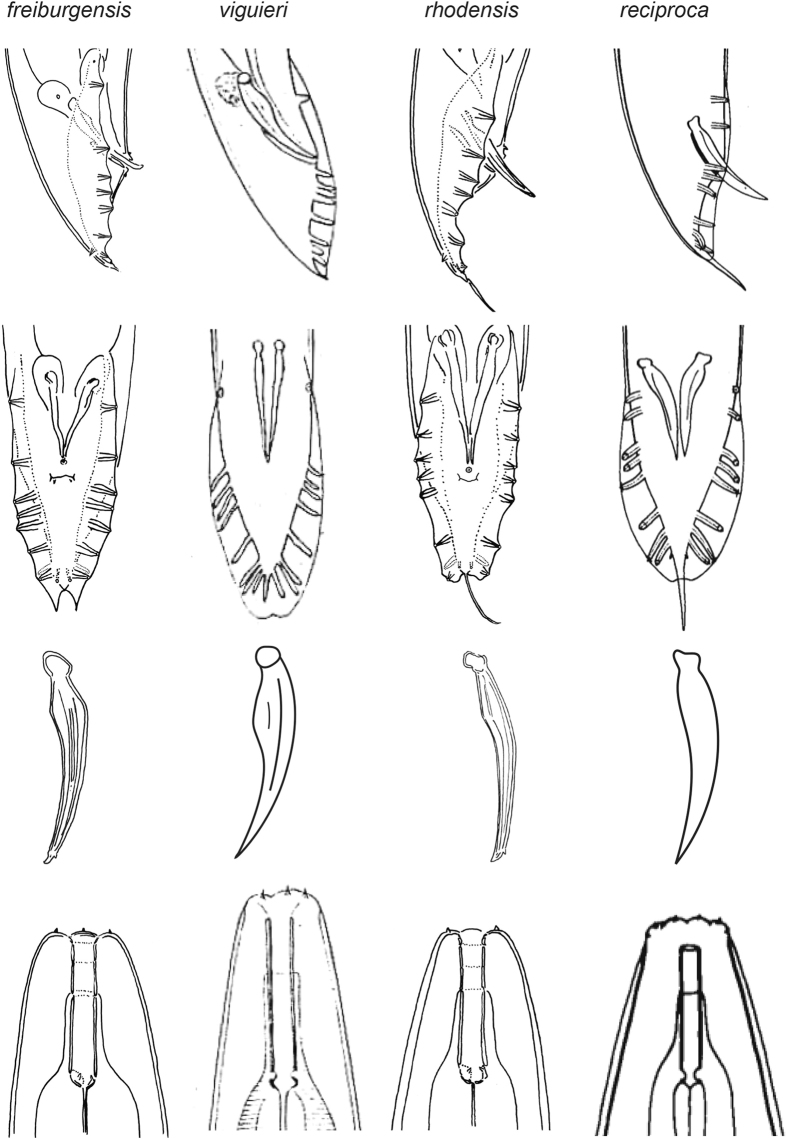
Male tail in ventral and lateral orientation, spicule (lateral) and stoma of four *Auanema* species. Images for *A. reciproca* are modified after Sudhaus^38^, for *A. viguieri* after Maupas^23^. Please note that the drawings were made by different authors and that the nematodes may have been under different culture conditions, or may have been fixed. The drawings are not to scale.

As discussed above, Molecular data strongly support the close relationship of *A. rhodensis* n. sp. and *A. freiburgensis* n. sp., but no such information is available for the other three species that we place in *Auanema*. However, the species in the genus share phenotypic characters, most notably a similar arrangement of bursal papillae. There are only 8 GPs of which the 6^th^ (counted from anterior) stands in an isolated position and the terminal two are grouped tightly with the more or less strongly papilliform phasmids. *A. viguieri* is described with 9 pairs of bursal papillae, and phasmids are not identified. However, given the number of other characters that *A. viguieri* has in common with *A. freiburgensis* and *A. rhodensis* (see below and Table 1), it is likely that the last pair of bursal papillae is also a pair of papilliform phasmids. The dauer juveniles of all species display tube waving behavior, although this information is not available for *A. seurati* ^39^. *A. rhodensis* n. sp., *A. freiburgensis* n. sp. and *A. viguieri* share additional phenotypic characters: the lips are fused into three groups of two; the median pharynx bulb is large and round; there are three sexes present, males, females and hermaphrodites. Whether the latter is also true for *A. reciproca* and *A. seurati* is unknown, but Sudhaus, 1974^38^ assumed that the females of *A. reciproca* reproduce by parthenogenesis (Table 1, Figs 6 and 7).

*A. rhodensis* n. sp. and *A. reciproca* are most similar to one another and are distinguished by a thick cuticle, a leptoderan tail, relatively long spicules with a square head (manubrium), a small distance between the first two GPs, similar to the distance of other GPs (GP1 is further anterior in *A. freiburgensis*. n. sp. and *A. viguieri*). *A. seurati* shares the thick cuticle and a similar arrangement of GPs, but here, the male tail is peloderan at least in some specimens. *A. rhodensis* n. sp. and *A. reciproca* differ from one another in the arrangement of GPs3–5, which form a group in *A. reciproca* that is separate from GP2, whereas the distances between GPs2–5 are similar in *A. rhodensis*.

*A. viguieri* differs from all other species by having only one pair of GPs situated precloacally. Females/hermaphrodites are longer and thinner (larger *a* value in Table 1) and their tails are slightly longer (smaller *c* value in Table 1).

*A. freiburgensis* n. sp. is unique in the presence of two triangular flaps at the distal end of the bursa. The eggs produced by hermaphrodites are larger than eggs in the other species.

### Biology

*A. rhodensis* females, males and hermaphrodites are produced by self-fertilizing hermaphrodites and by mated females^14, 22^. Sex determination between males and non-males is chromosomal, as indicated by the presence of an unpaired X chromosome during meiosis in the male germline^21^. About 10% of the progeny by a selfing hermaphrodite is male^14, 22^, presumably as result of X chromosome non-disjunction during gametogenesis. Cross-progeny between *A. rhodensis* males and females is less than 5% male^14, 21^. This occurs because males produce mostly functional X-bearing gametes (contributing to female offspring) and non-functional nullo-X cells^21^.

Hermaphrodites that underwent more than fifty rounds of inbreeding still produce females and hermaphrodites by selfing^22^. This result suggests that the distinction between hermaphrodites and females is non-genetic. *A. rhodensis* hermaphrodites and females tend to produce a higher proportion of female and male progeny in the first three days of adulthood^22^. Hermaphrodite and female L2 juveniles can be distinguished by the size of their gonad primordium^14^, which is larger in female juveniles^19^. Hermaphrodites always undergo dauer development, independently of the environmental conditions^14, 22^. When in the presence of food, they remain in this stage for about 24 hours and then resume development. L2 juveniles with large gonad primordium, fated to become females, can be forced towards dauer development by the removal of the hormone precursor cholesterol^22^. Under those conditions, the juveniles develop into hermaphrodites. Inhibition of dauer development by the addition of the hormone dafachronic acid in small-sized gonad primordium animals results in female development. Thus, the dauer stage is necessary and sufficient for hermaphrodite development in *A. rhodensis* ^22^.

*A. freiburgensis* hermaphrodites, when in isolation, produce mostly females and males (91.1% females, 8.7% males, 0.2% hermaphrodites; n = 1,950 progeny from 9 hermaphrodites). In contrast, hermaphrodites collected from crowded plates produce mostly hermaphrodites (6.2% females, 13.6% males, 80.1% hermaphrodites; n = 675 progeny from 6 hermaphrodites). Similar to *A. rhodensis* males, *A. freiburgensis* males mated with females sire cross-progeny with noticeably skewed sex ratios (18% males, n = 1,025 F1s, 8 crosses). Hermaphrodite and female L2 juveniles cannot be distinguished by the size of their gonad primordium. Juveniles that pass through the dauer stage always become hermaphrodite adults.

#### Dauer juveniles

In both species, the dauer juveniles display the unusual tube waving behavior that was first described by Osche^49^: Dauer juveniles do not immediately shed the J2 cuticle. This cuticle is instead attached to the substrate and opens at the anterior end so that the dauer juvenile partially protrudes from this tube-like sheath while waving. When disturbed, the dauer juvenile retracts back into the tube.

## Discussion

The evolution of two features observed in *Auanema* species merits further discussion. First, the two new species described here and *A. viguieri* were found to be trioecious, with males and females and hermaphrodites. A reproductive system with these three sexes is also present in the insect pathogenic *Heterorhabditis* species. As in *A. rhodensis* and *A. freiburgensis*, *Heterorhabditis bacteriophora* development into hermaphrodites is determined by the passage through the dauer stage^19, 50^. Our current as well as previous phylogenetic analyses^16, 37^ clearly show that *Auanema* n. gen. and *Heterorhabditis* are not closely related. Another clade with a trioecious reproductive system is Rhabdiasidae^51^. These parasites of amphibians are only distantly related to *Auanema* n. gen. or *Heterorhabditis*. Thus, a trioecious reproductive system evolved three times within Nematoda. Interestingly, in each case, only dauer juveniles (homologous infective larvae in Rhabdiasidae) develop into hermaphrodites. Since the dauer juvenile is the dispersal stage in these species, being hermaphroditic conveys an obvious advantage. A single hermaphroditic individual can establish a population by self-fertilization. This feature may thus be selected for. How the decision between female and hermaphrodite development is regulated on a mechanistic level is insufficiently understood in all three lineages^19^, precluding speculations on parallel or convergent evolution of this trait.

The co-existence of males, females and hermaphrodites has been considered an evolutionarily transient state^52^, which may explain the rarity of this mating system in animals. In the case of nematodes, it is not clear if trioecy evolved from dioecy or from androdioecy. In *Caenorhabditis*, at least two mutations must occur to convert a female into a hermaphrodite^53^. In contrast, only one mutation is sufficient to convert a hermaphrodite from an androdioecious mating type into a female^54, 55^. Although these two scenarios are relatively easy to postulate, it is more difficult to explain how the three sexes could co-exist because hermaphrodites easily outcompete females^54, 55^.

In *Auanema* n. gen. species, the evolution of trioecy might have occurred in a different way than in *Caenorhabditis*, because hermaphrodites and females are genetically identical^19^. Although speculative at this point, the existence of built-in mechanisms to generate females and hermaphrodites that are not dependent on the frequency of specific alleles may make trioecy more stable. *A. rhodensis* developmental decision to generate different XX progeny seems to be determined by an age-specific maternal factor^22^. The highest proportion of female progeny is produced by young (1–3 day old) mothers, while older mothers produce mostly hermaphrodite progeny. To understand how hermaphroditism evolved in *Auanema*, it would be useful to discover the nature and regulation of the maternal factor, and how dauer development is coupled to hermaphrodite development. It is possible that cellular memory of early developmental history influences L4 and adult gene expression. A similar phenomenon occurs in *C. elegans* ^56^, in which phenotypic differences have been detected between animals that bypassed the dauer stage and animals that passed through dauer.

The second remarkable characteristic of *Auanema* n. gen. species is the tube waving behaviour of the dauer juveniles. This behaviour was first observed by Osche^49^ for *Rhabditella* species and *A. viguieri* and then discovered in *A. reciproca* ^24^. Because these species were thought to be unrelated, tube waving was assumed to have evolved three times independently^38, 40, 57^. However, our phylogenetic analysis clearly shows that the species with tube waving behaviour (*Rhabditella* spp., *Cephaloboides* sp. and *Auanema* species) form a clade. Therefore, a more parsimonious interpretation is that tube waving evolved only once. The biological and ecological function of tube waving is unknown. It has been speculated that the tube may serve to protect the dauer juvenile from predators or desiccation or that this waving behaviour enables the dauer juvenile to choose a specific phoretic carrier^57^.

## Electronic supplementary material

Supplementary info
