# Supplementary material for: Description of two three-gendered nematode species in the new genus Auanema (Rhabditina) that are models for reproductive mode evolution
Source: Sci Rep. 2017 Sep 11;7:11135. doi: 10.1038/s41598-017-09871-1 (PMC5593846; doi:10.1038/s41598-017-09871-1)
Supplement: Supplementary file 1 — Supplementary info [file 41598_2017_9871_MOESM1_ESM.doc]

**Description of two three-gendered nematode species in the new genus *Auanema* (Rhabditina) that are models for reproductive mode evolution**

Natsumi Kanzaki^1#^, Karin Kiontke^2#^, Ryusei Tanaka^1,3^, Yuuri Hirooka^1,4^, Anna Schwarz^5^, Thomas Müller-Reichert^5^, Jyotiska Chaudhuri^6^, Andre Pires-daSilva^7^*

^1^*Forest Pathology Laboratory, Forestry and Forest Products Research Institute, 1 Matsunosato, Tsukuba, Ibaraki 305-8687 Japan.* E-mail: *nkanzaki@ffpri.affrc.go.jp*

^2^ *Department of Biology, New York University, 100 Washington Square E., New York, NY, 10003, USA. E-mail:* [*kk52@nyu.edu*](mailto:kk52@nyu.edu)

^3^*Present address:* *Division of Parasitology, Faculty of Medicine, University of Miyazaki, Miyazaki 889-1692, Japan. E-mail: caenorhabditisjaponica@gmail.com*

^4^*Present address: Department of Clinical Plant Science, Faculty of Bioscience and Applied Chemistry, Hosei University, Kajino-cho 3-7-2, Koganei, Tokyo 184-8584, Japan. E-mail:* [*yuuri.hirooka.54@hosei.ac.jp*](mailto:yuuri.hirooka.54@hosei.ac.jp)

*^5^Experimental Center, Medical Faculty Carl Gustav Carus, Technische Universität Dresden, Fiedlerstraße 42, 01307 Dresden, Germany. Email: mueller-reichert@tu-dresden.de*

*^6^Buck Institute for Research on Aging, 8001 Redwood Blvd, Novato, CA 94945, USA. E-mail: JChaudhuri@buckinstitute.org*

*^7^School of Life Sciences, University of Warwick, Coventry, UK. Email:* [*andre.pires@warwick.ac.uk*](mailto:andre.pires@warwick.ac.uk)

# equal contribution

**Corresponding author*

**Supplementary Figures**

**
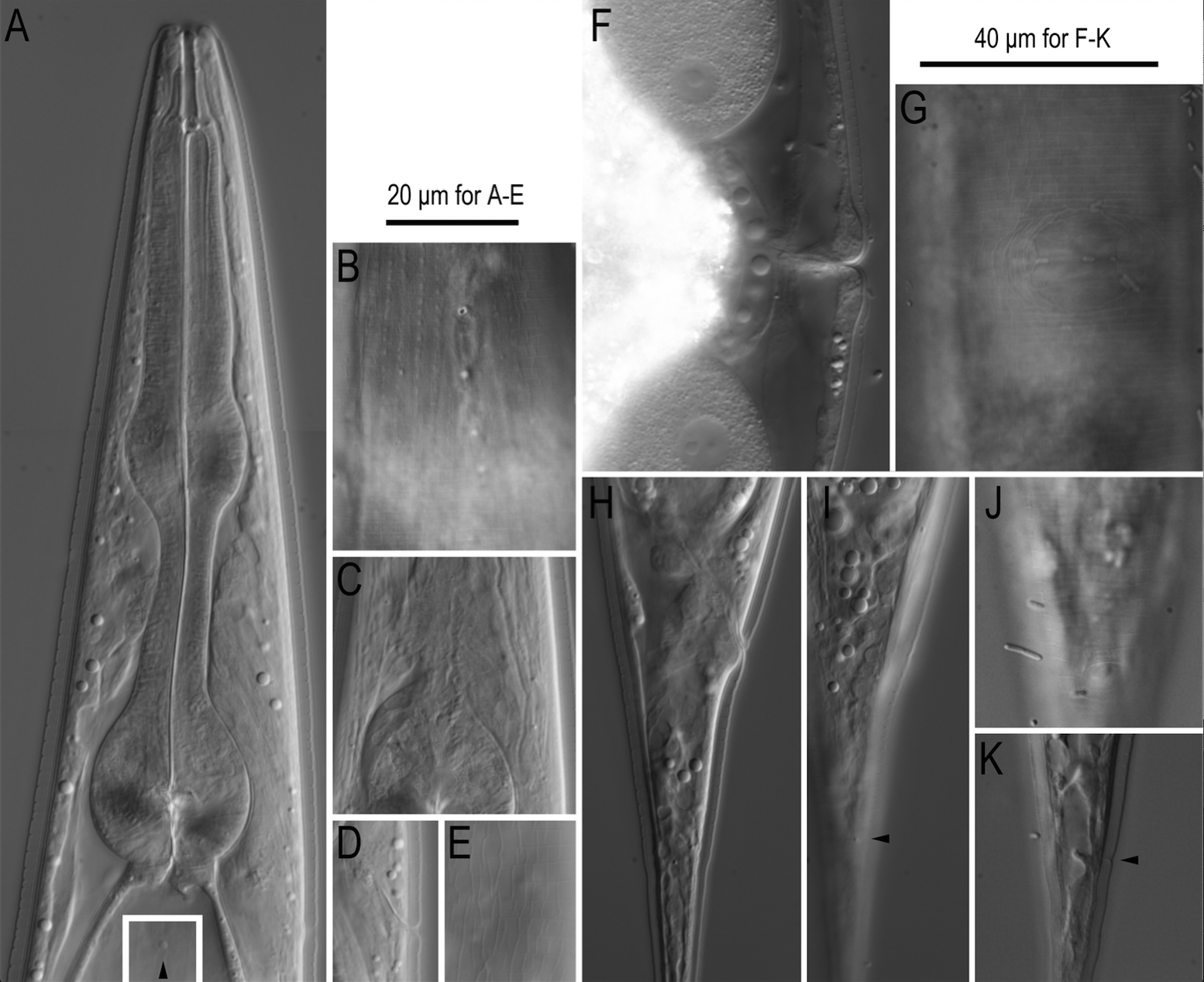
**

**Supplementary FIG. 1.** Adult hermaphrodite of *A. rhodensis* n. gen., n. sp. A: Anterior part in right lateral view (deirid on the body surface is enclosed in square at the corresponding position of the body, and indicated with an arrowhead, because the focal plane for the other part is in the middle of the worm); B: Excretory pore in ventral view; C: Same animal as in A at a focal plane showing the nerve ring; D: Same animal as in A at a focal plane showing the excretory pore; E: Body surface structure at mid body in lateral view; F : Vulval opening in right lateral view; G: Vulval opening in ventral view; H: Anal region in right lateral view; I: Same animal as in H at a focal plane showing phasmid opening (arrowhead); J: Anal opening in ventral view; K: Phasmid in ventral view.


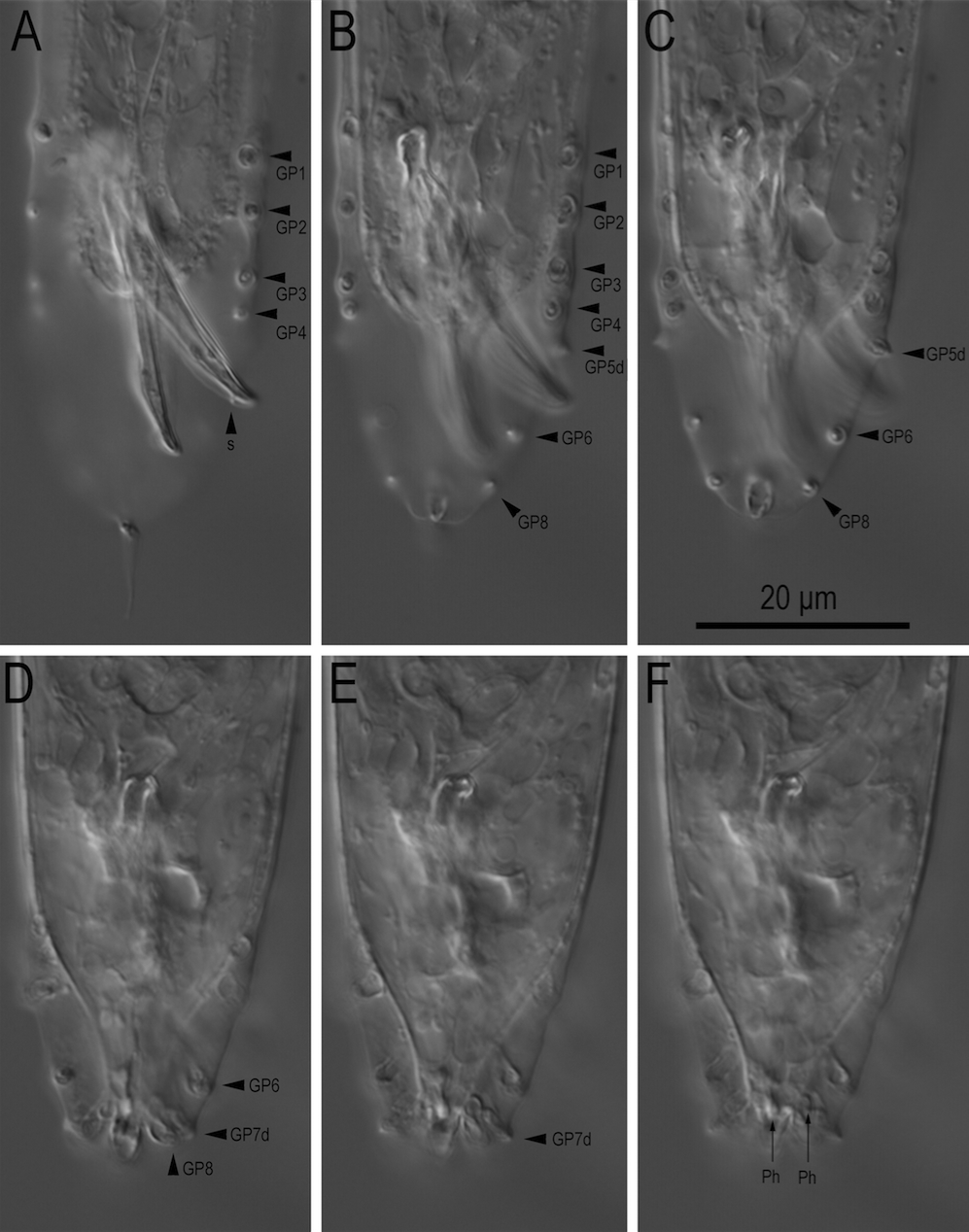


**Supplementary FIG. 2.** Male tail of *A. rhodensis* n. gen., n. sp. in ventral view in six different focal planes. Genital papillae (GP#) and dorsal spike (thorn) of spicule (s) are indicated with arrowheads, and phasmids are indicated with small arrows.


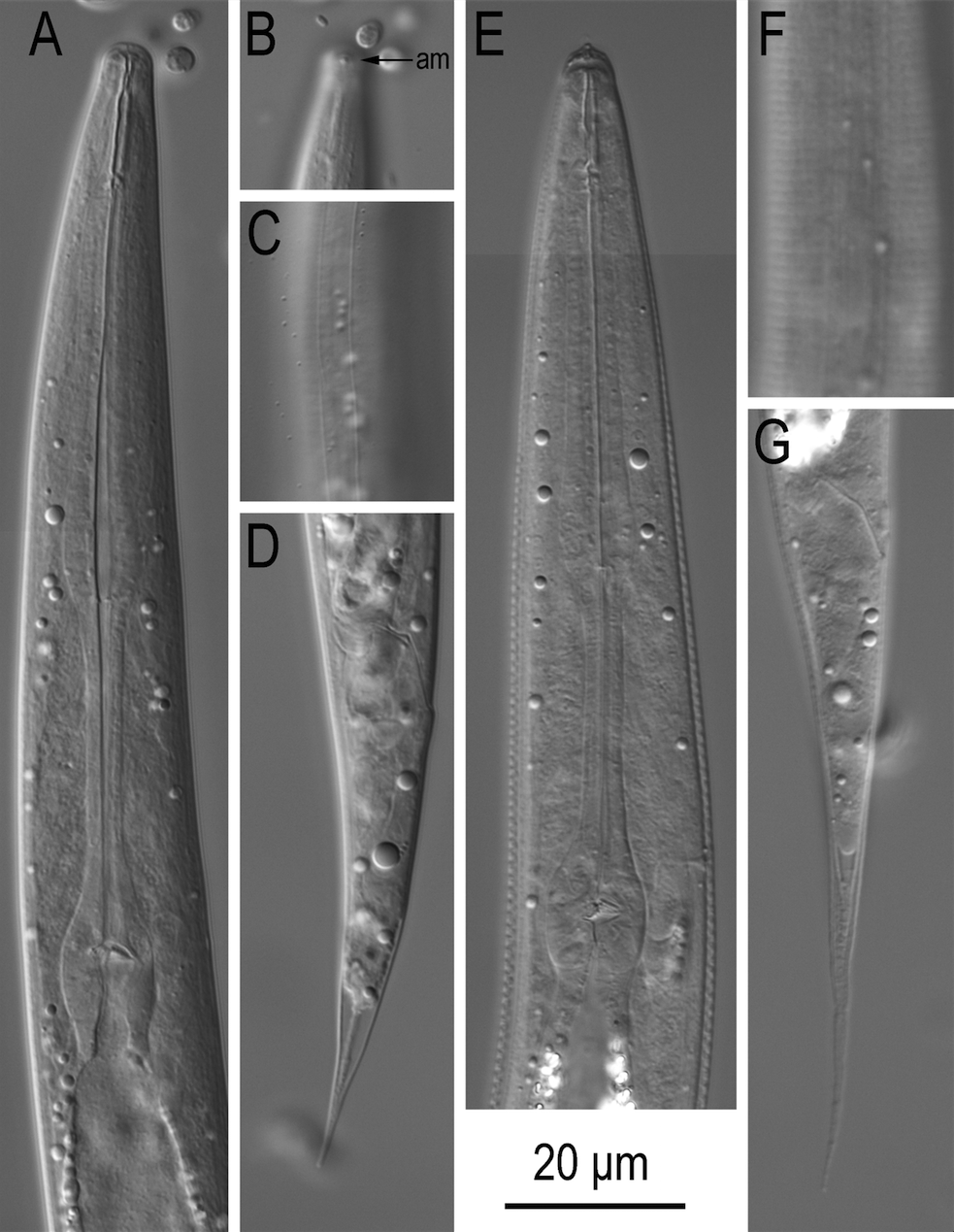


**Supplementary FIG. 3.** Dauer juveniles of *A. rhodensis* n. gen., n. sp. A, B: Anterior region of a waving individual in different focal planes in right lateral view (AM: amphid); C: Body surface of a waving individual; D: Tail of a waving individual in right lateral view; E: Anterior part of an ensheathed individual in in right lateral view; F: Body surface (J2 cuticle) of an ensheathed individual; G: Tail of an ensheathed individual in right lateral view.


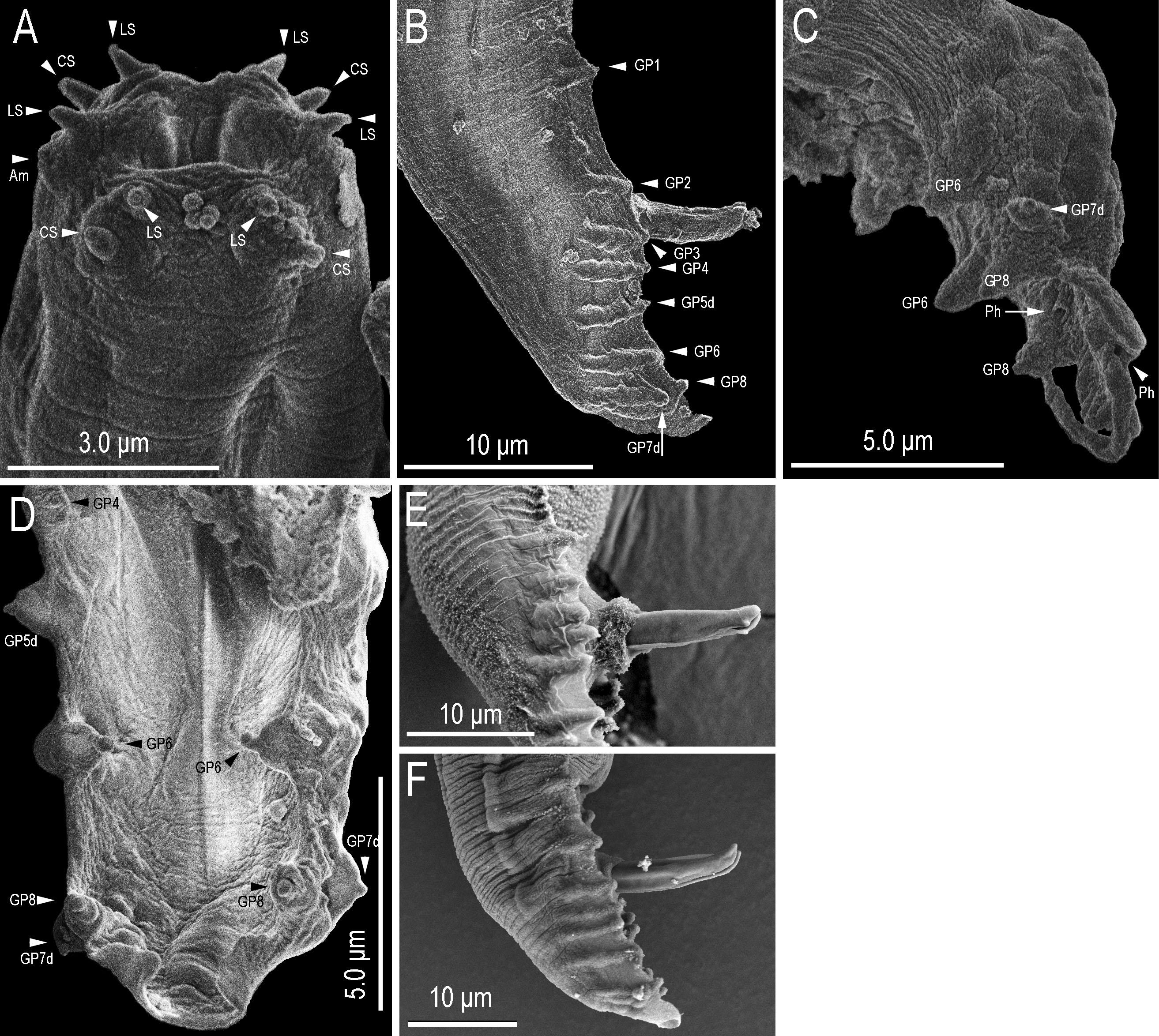


**Supplementary FIG. 4.** Scanning electron micrographs of *Auanema* n. gen. spp. A: Male cephalic region of *A. rhodensis* n. gen., n. sp. (Am: amphid; LS: labial sensillum; CS: cephalic sensillum); B: Male tail of *A. freiburgensis* n. gen., n. sp. in right lateral view (Genital papillae are indicated with arrowheads); C: Male tail tip of *A. rhodensis* n. gen., n. sp. in left lateral view (Genital papillae and phasmids are indicated with arrowheads); D: Male tail of *A. freiburgensis* n. gen., n. sp. in ventral view (Genital papillae are indicated with arrowheads); E: Male tail of *A. rhodensis* n. gen., n. sp. in lateral view showing a small dorsal spike-like projection near the tip of each spicule. F. Male tail of *A. freiburgensis* n. gen., n. sp in lateral view.


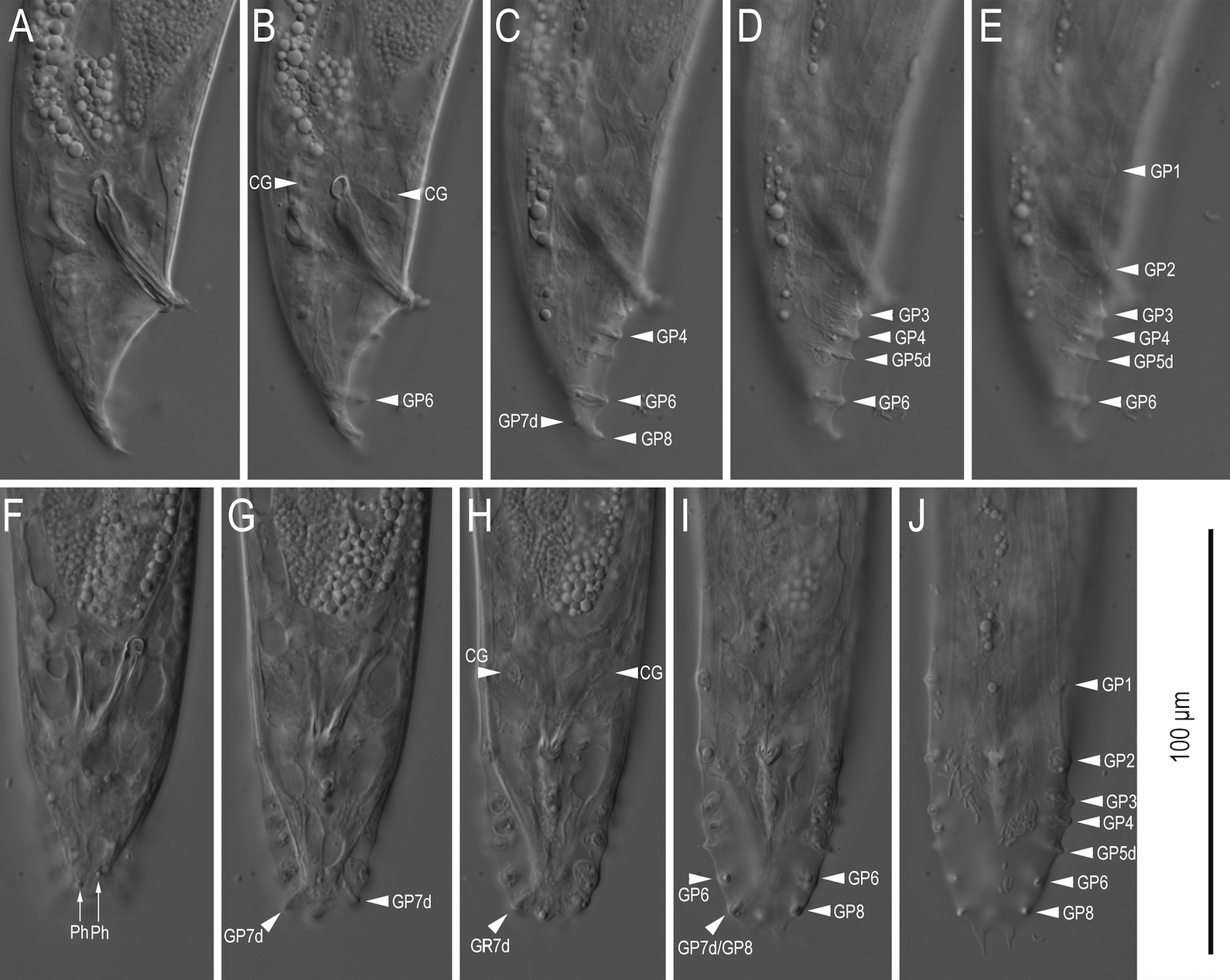


**Supplementary FIG. 5.** Male tail of *A. freiburgensis* n. gen., n. sp. in different focal planes. A-E: Right lateral view; F-J: Ventral view. Genital papillae (GP#) and cloacal (rectal) glands (CG) are indicated with arrowheads, and phasmids are indicated with small arrows.

**Supplementary Data 1**

GenBank Accession numbers for the sequences of the three genes used to reconstruct the phylogeny.

| Strain | SSUrDNA | LSUrDNA | RNA Polymerase II |
| --- | --- | --- | --- |
| *Auanema freiburgensis* SB372 | KY680647 | KY680646 | KY646107 |
| *Auanema rhodensis* n.sp. SB347 | EU196004 | EU195960 | EU153428 |
| *Caenorhabditis elegans* | EU196001 | X03680 | M29235 |
| *Cephaloboides* sp. SB363 | EU196005 | EU195961 | EU178801 |
| *Choriorhabitis dudichi* SB122 | AF083012 | EU195975 | EU161965/EU153409 |
| *Heterorhabditis* sp. | AF036593 | EU195993 | ACKM01001433.1 |
| *Haemonchus* sp. | L04152.1 | AM039742.1 | *H. contortus* genome at Sanger* |
| *Litoditis mediterranea* | AF083020 | EU195973 | EU161960/EU153417 |
| *Mesorhabditis longespiculosa* DF5017 | EU196014 | EU195980 | EU153435 |
| *Oscheius tipulae* CEW1 | EU196009 | EU195969 | EU153430/EU153414 |
| *Poikilolaimus oxycercus* SB200 | AF083023 | EU195984 | EU161976 |
| *Pristionchus pacificus* PS312 | AF083010 | EU195982 | EU161964/ EU153426 |
| *Prodontorhabditis wirthi* DF5074 | AY602179 | AY602169 | AY604469 |
| *Rhabditella axei* DF5005 | U13934 | AY602177 | AY602183 |
| *Metarhabditis blumi* DF5010 | U13935 | EU195965 | EU161970/EU153412 |
| *Rhabditoides inermis* | AF082996 | EU195981 | EU161963/ EU153425 |
| *Teratorhabditis palmarum* DF5019 | U13937 | EF990717 | EU15343/U73455 |

* Assembled super contigs 12/6/12 scaffold427|size159813.2.25081

**Supplementary Data 2**

1. movies showing Z planes of the following structures of the new species. High-resolution movies deposited in https://doi.org/10.6084/m9.figshare.5341372:

*A. rhodensis*

| Hermaphrodite stoma lateral right view | https://youtu.be/7wyKT6DdtrM |
| --- | --- |
| Hermaphrodite pharynx lateral right view | https://youtu.be/02bF7HQJwJc |
| Hermaphrodite vulva region lateral right view | https://youtu.be/9yOWaou3Ai0 |
| Hermaphrodite tail lateral right view | https://youtu.be/oP3hg1YzYAY |
| Female stoma lateral left view | https://youtu.be/EvWIQ-JPTYk |
| Female pharynx lateral left view | https://youtu.be/PLy8IIbhYao |
| Female vulva region lateral left view | https://youtu.be/7Ln4LD-rY70 |
| Female tail ventral view | https://youtu.be/OzFEElId7p0 |
| Male stoma lateral left view | https://youtu.be/CcRltuzhYqg |
| Male pharynx lateral left view | https://youtu.be/FUvBOw3sTN8 |
| Male tail ventral view | https://youtu.be/79YxpGWFfTA |
| Male tail lateral view | https://youtu.be/nQnoMGpR5jo |
| Male macerated spicules | https://youtu.be/oMNlQmtRRr0 |

*Auanema freiburgensis*

| Hermaphrodite stoma lateral right view | https://youtu.be/oN66N2D3v4g |
| --- | --- |
| Female pharynx lateral right view | https://youtu.be/K6elQauXd-0 |
| Hermaphrodite anterior gonad | https://youtu.be/0tgPkGdsZVw |
| Hermaphrodite tail lateral left view | https://youtu.be/kjeWdTxKtkg |
| Female stoma sublateral left view | https://youtu.be/_YFvB9DpoAU |
| Female tail with anus and phasmids lateral right view | https://youtu.be/n2owMz-I5k8 |
| Male stoma lateral right view | https://youtu.be/oGaUO-OFg10 |
| Male pharynx lateral left view | https://youtu.be/1xCM_9Y64Co |
| Male tail ventral view | https://youtu.be/TTdtetAbtJo |
| Male tail lateral view | https://youtu.be/j8-J2QsHc4A |
| Male macerated spicules | https://youtu.be/jCp4XvAqcfY |
| Dauer larva stoma lateral right view | https://youtu.be/FTjqUJ9z_As |
| Dauer larva pharynx lateral left view | https://youtu.be/F8Isg1xg2-Q |
| Dauer larva gonad primordium ventral view | https://youtu.be/1YrI3gxRbb0 |

**Supplementary Table 1. Measurements. Given as range (average) in µm.**

(L= body length; a = L/maximum body width; b = L/pharynx length; c = L/tail length; c’ tail length/ABD; ABD = anal body diameter; V = vulva position in percent of L)

|  | ***Auanema freiburgensis* n. sp. (SB372)** | | | | ***Auanema rhodensis* n. sp. (SB347)** | | | |
| --- | --- | --- | --- | --- | --- | --- | --- | --- |
| Character | male | hermaphr. | female | dauer | male | hermaphr. | female | dauer |
| n | 10 | 10 | 10 | 10 | 10 | 10 | 10 | 10 |
| L | 625-705 (686) | 977-1135 (1054) | 969-1110 (1035) | 358-389 (373) | 595-737 (665) | 767-1401 (1108) | 980-1214 (1096) | 388-410 (400) |
| a | 16.4-17.9 (17.0) | 14.4-18.5 (16.2) | 18.8-21.4 (20.3) | 16.4-19.6 (18.2) | 11.2-19.0 (16.6) | 13.8-21.5 (17.3) | 13.7-22.6 (17.0) | 18.0-21.3 (19.5) |
| b | 5.4-6.1 (5.9) | 7.4-8.5 (8.0) | 7.8-9.1 (8.3) | 3.5-3.9 (3.7) | 4.0-5.1 (4.7) | 5.8-7.8 (7.0) | 6.9-8.7 (7.8) | 3.5-3.8 (3.7) |
| c | 22.2-26.0 (23.4) | 5.1-6.0 (5.5) | 5.4-5.9 (5.7) | 10.2-13.1 (11.7) | 16.7-32.3 (23.6) | 4.3-12.2 (8.6) | 5.1-6.3 (5.6) | 8.7-11.7 (9.9) |
| c’ | 1.9-2.1 (2.0) | 9.4-11.6 (10.2) | 9.8-10.6 (10.3) | 4.8-6.5 (5.6) | 1.2-2.2 (1.7) | 4.5-8.3 (6.3) | 8.2-12.5 (10.0) | 4.9-7.0 (6.0) |
| V | -- | 44.7-50.5 (48.1) | 40.1-45.5 (43.2) |  | -- | 39.4-50.1 (47.2) | 44.0-50.0 (48.1) | -- |
| Body diam. | 38-43 (40) | 53-72 (65) | 45-58 (51) | 18.5-23.0 (20.6) | 36-54 (41) | 50-78 (64) | 49-76 (66) | 18.6-22.7 (20.6) |
| Lip region diam. | 5.8-7.6 (6.6) | 6.4-9.1 (7.9) | 7.4-9.5 (8.1) | 3.0-4.7 (3.9) | 9.7-11.6 (10.5) | 9.4-13.7 (11.0) | 9.7-13.2 (11.3) | 4.0-5.7 (4.7) |
| Stoma length | 13.2-14.9 (14.0) | 15.9-18.3 (17.3) | 14.6-15.9 (15.2) | 6.5-8.7 (7.4) | 12.5-14.9 (13.6) | 12.4-16.4 (14.2) | 12.3-13.6 (12.9) | 7.2-9.1 (8.1) |
| Stoma diam. | 1.5-1.8 (1.7) | 1.8-2.6 (2.3) | 2.0-3.1 (2.5) | 1.0-2.1 (1.4) | 1.2-1.8 (1.5) | 1.9-2.4 (2.1) | 1.8-2.1 (2.0) | 1.0-2.1 (1.4) |
| Pharynx length | 106-115 (111) | 119-131 (126) | 117-123 (119) | 90-101 (95) | 127-141 (134) | 130-159 (140) | 127-147 (136) | 96-111 (101) |
| Corpus length | 57-63 (59) | 60-68 (64) | 50-62 (56) | -- | 68-84 (73) | 66-84 (74) | 66-87 (75) | -- |
| Median bulb diam. | 12.8-16.5 (15.0) | 19.6-25.7 (22.5) | 17.4-20.1 (18.6) | -- | 16.8-20.3 (18.5) | 18.6-25.6 (23.0) | 18.7-26.1 (22.3) | -- |
| Terminal bulb diam. | 16.2-20.5 (17.9) | 23.4-29.2 (25.9) | 19.8-23.4 (21.5) | 6.9-10.6 (8.8) | 19.9-24.5 (22.4) | 26.8-28.7 (27.6) | 20.8-28.1 (24.4) | 6.8-10.0 (8.5) |
| Anterior end to excretory pore | 108-118 (113) | 133-140 (136) | 117-122 (118) | -- | 120-135 (130) | 106-135 (123) | 113-139 (128) | -- |
| Gonad length | 387-420 (401) | 446-516 (478) | 468-552 (521) | 15.5-19.4 (17.4) | 325-411 (379) | 347-510 (400) | 358-535 (444) | 18.6-23.6 (21.0) |
| Anterior gonad branch | -- | 221-260 (236) | 211-231 (220) | -- | -- | 145-231 (194) | 180-268 (222) | -- |
| Posterior gonad branch | -- | 223-279 (241) | 257-326 (301) | -- | -- | 151-312 (206) | 178-275 (222) | -- |
| Length of flexure | 96-107 (100) | -- | -- | -- | 82-106 (93) | -- | -- | -- |
| Egg length | -- | 58-61 (60) | 43-51 (48) |  | -- | 43-54 (48) | 39-48 (43) | -- |
| Egg width | -- | 29-38 (34) | 14-21 (17) |  | -- | 22-37 (28) | 24-32 (28) | -- |
| Tail length | 25.6-31.8 (29.4) | 187-198 (191) | 169-190 (182) | 27.5-36.8 (32.1) | 19.6-35.6 (28.9) | 96-177 (134) | 169-218 (196) | 34.8-46.5 (40.7) |
| Anus to phasmids/ABD | -- | 1.5-2.2 (1.9) | 1.4-1.7 (1.5) | -- | -- | 1.7-2.4 (2.0) | 1.1-1.3 (1.2) | -- |
| ABD | 12.9-16.7 (14.9) | 16.5-20.2 (18.8) | 15.9-19.1 (17.7) | 4.8-6.9 (5.7) | 15.6-18.1 (17.1) | 17.8-25.8 (21.4) | 16.5-21.2 (19.7) | 5.9-7.4 (6.8) |
| Rectum length | -- | 21.5-29.1 (25.6) | 19.8-26.1 (23.9) | -- | -- | 20.5-30.0 (24.6) | 18.9-25.4 (23.1) | -- |
| Spicules | 20.8-23.4 (22.2) | -- | -- | -- | 28.9-31.6 (30.5) | -- | -- | -- |
| Gubernaculum | 9.7-13.9 (11.0) | -- | -- | -- | 12.6-14.9 (13.8) | -- | -- | -- |
